# Tongue image quality assessment based on a deep convolutional neural network

**DOI:** 10.1186/s12911-021-01508-8

**Published:** 2021-05-05

**Authors:** Tao Jiang, Xiao-juan Hu, Xing-hua Yao, Li-ping Tu, Jing-bin Huang, Xu-xiang Ma, Ji Cui, Qing-feng Wu, Jia-tuo Xu

**Affiliations:** 1 Basic Medical College Shanghai University of Traditional Chinese Medicine, 1200 Cailun Road, Pudong New Area, Shanghai, 201203 China; 2 Shanghai Collaborative Innovation Center of Health Service in TCM, Shanghai University of TCM, 1200 Cailun Road, Shanghai, 201203 China; 3 School of Information Science and Engineering, Xiamen University, Xiamen, 361005 China

**Keywords:** Tongue diagnosis, Quality assessment, Deep learning, ResNet, DenseNet

## Abstract

**Background:**

Tongue diagnosis is an important research field of TCM diagnostic technology modernization. The quality of tongue images is the basis for constructing a standard dataset in the field of tongue diagnosis. To establish a standard tongue image database in the TCM industry, we need to evaluate the quality of a massive number of tongue images and add qualified images to the database. Therefore, an automatic, efficient and accurate quality control model is of significance to the development of intelligent tongue diagnosis technology for TCM.

**Methods:**

Machine learning methods, including Support Vector Machine (SVM), Random Forest (RF), Gradient Boosting Decision Tree (GBDT), Adaptive Boosting Algorithm (Adaboost), Naïve Bayes, Decision Tree (DT), Residual Neural Network (ResNet), Convolution Neural Network developed by Visual Geometry Group at University of Oxford (VGG), and Densely Connected Convolutional Networks (DenseNet), were utilized to identify good-quality and poor-quality tongue images. Their performances were made comparisons by using metrics such as accuracy, precision, recall, and F1-Score.

**Results:**

The experimental results showed that the accuracy of the three deep learning models was more than 96%, and the accuracy of ResNet-152 and DenseNet-169 was more than 98%. The model ResNet-152 obtained accuracy of 99.04%, precision of 99.05%, recall of 99.04%, and F1-score of 99.05%. The performances were better than performances of other eight models. The eight models are VGG-16, DenseNet-169, SVM, RF, GBDT, Adaboost, Naïve Bayes, and DT. ResNet-152 was selected as quality-screening model for tongue IQA.

**Conclusions:**

Our research findings demonstrate various CNN models in the decision-making process for the selection of tongue image quality assessment and indicate that applying deep learning methods, specifically deep CNNs, to evaluate poor-quality tongue images is feasible.

## Background

Tongue inspection has a long history as the most intuitive, simple and effective diagnostic method in Traditional Chinese Medicine (TCM)[1–3]. However, traditional tongue diagnosis is affected by factors such as the external environment and doctors’ subjective clinical experience. Computerized tongue diagnosis systems are gradually being accepted by an increasing number of clinicians as a medical application for the health assessment and diagnosis of diseases, such as type-2 diabetes mellitus [4–7], breast cancer [8], colorectal cancer [9], appendicitis [10], and gastritis [11].

**Fig. 1:**
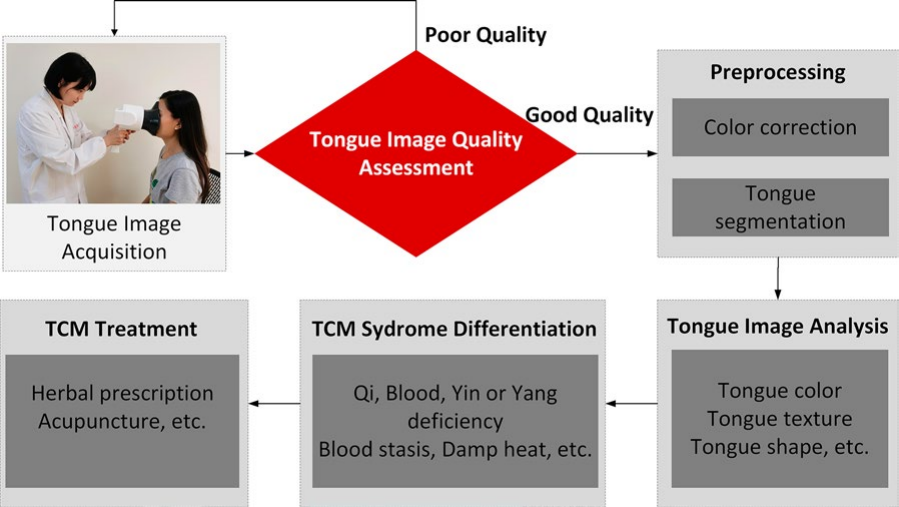
Overview of intelligent TCM tongue diagnosis procedures

Research teams worldwide have carried out more than 20 years of objective research on tongue diagnosis, but no standard tongue image dataset with large samples has been established. The quality of tongue images is the basic component to construct standard datasets in the field of TCM tongue diagnosis. With the popularization of the clinical application of digital tongue pictures, massive tongue image data are produced. The quality of tongue images is an important prerequisite for the clinical application of tongue diagnosis [1, 3]; see Fig. 1.

**Fig. 2:**
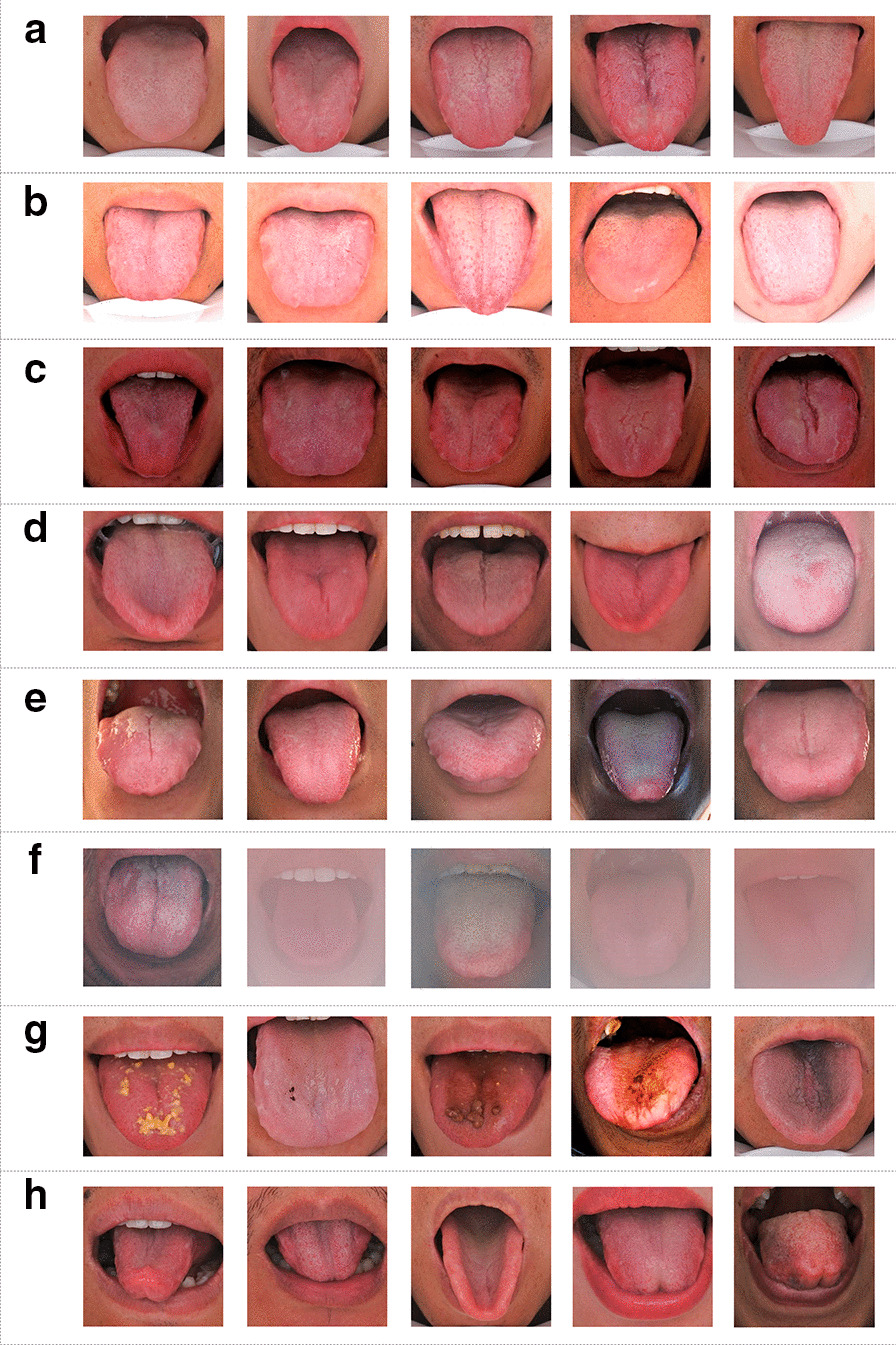
Good-quality versus poor-quality tongue images. **a** Good-quality tongue images; **b**–**h** poor-quality tongue images: **b** overexposed tongue images; **c** underexposed tongue images; **d** blurry tongue images; **e** tongue images with light leakage; **f** foggy tongue images; **g** stained tongue images or tongue coating; **h** tongue images with unsuitable posture.

Good-quality tongue images should have the following characteristics: ① tongue extended and stretched to the outside of the lower lip; ② no food residue on the tongue or stained tongue coating; ③ normal exposure; ④ no blurring caused by tongue movement in the process of recording; ⑤ no light leakage; and ⑥ no blurring caused by breath condensation on the camera lens, as shown in Fig. 2a. We found that in the process of using tongue diagnosis equipment, despite standardized tongue image acquisition training, abnormal tongue images are still common in the clinical tongue image acquisition process, mainly from two aspects: operators and participants.

There are 7 types of tongue images with poor quality, including those with light leakage, overexposure, underexposure, blurry focus, stained tongue coating, fog, and incorrect tongue extension posture, as shown in Fig. 2b–h. Overexposure causes a tongue image to be brighter and the tongue colour to be whiter, as shown in Fig. 2b. Underexposure makes the tongue look darker in the image, and the tongue colour tends to be dark red and crimson, as shown in Fig. 2c. Shaking or vibration of the tongue during the shooting process results in blurry focus of the tongue images, as shown in Fig. 2d. Light from the outside of the instrument can enter the inside of the instrument through a gap (such as the gap between the face and the tongue diagnostic instrument cover), and then the brightness of some areas of the tongue image becomes too bright, as shown in Fig. 2e. Foggy tongue images are caused by the subject's exhalation condensing in the camera during the shooting process, resulting in the tongue images shown in Fig. 2f. During the shooting, food residue remained on the subject's tongue. Foreign objects on the tongue obscured characteristic information such as the colour and texture of the tongue itself, as shown in Fig. 2g. During the shooting, some subjects did not hold the tip of their tongue away from their lips, the tip of their tongue was upturned, or the extension of their tongue was uneven, which obscured the colour, texture and other information in some areas of their tongue, as shown in Fig. 2h.

All poor-quality tongue images affect the analysis of the colour, shape, texture of the tongue image and directly lead to a wrong diagnosis of the patient's TCM syndromes, causing severe interference with the development of intelligent diagnosis technology of TCM tongue diagnosis and the accuracy of TCM clinical remote diagnosis and treatment.

Image quality assessment (IQA) mainly evaluates the quality of images. Both manual and automatic methods can be used to evaluate image quality. At present, the main approach to removing pictures in these situations is manual. The manual method is based on TCM diagnosis and clinical experts' perception assessment of the quality of tongue images. Zhang et al. proposed new features for recognizing poor-quality and good-quality tongue images, including color features, textures spectral features, spatial and spectral entropies features [12, 13]. The proposed features were manually extracted from tongue images, and then were fed onto SVM and RF for binary classification of tongue IQA, respectively. Their results of accuracy are nearly 90%. The proposed methods by [12, 13] only focused on two kind of poo-quality tongue images. This method is costly, labour intensive, error prone and inefficient and cannot be automated in real time. Therefore, an efficient and accurate quality control model of tongue images is essential for the clinical use of tongue diagnosis instruments. This research focused on solving the quality control problem of tongue images and automatically removing poor-quality tongue images.

In recent years, with the tremendous success of deep convolutional neural networks (CNNs) and the development of deep learning algorithms, the classification accuracy and efficiency of image analysis technology based on CNNs have been dramatically improved. These networks have been widely used in image segmentation, image classification, face recognition, etc., and has become the current mainstream algorithm [14–16]. CNNs, a representative deep learning method, have gradually become a research hotspot in the field of objective tongue diagnosis.

In general, CNN architectures can avoid feature selection manually and automatically extract features, which are key elements to enable the intelligent tongue diagnosis system into TCM clinical practice. Although several previous studies have reported encouraging results using CNN methods to extract tongue image features for tongue colour (tongue body and tongue coating) classification [17–19], tongue image characteristic recognition (tooth-marked tongue [20–22], tongue cracking [23]), tongue image segmentation [24–31], and clinical application in herbal medicine [32, 33], they usually ignore the quality of tongue images or implicitly assume the good quality of tongue images. Thus, the medical application of deep learning methods to the field of tongue diagnosis has not achieved much so far.

In this research, we focus on the model construction method of automatically rejecting unqualified tongue images based on a deep CNN model to evaluate the quality of tongue images.

## Methods

### Tongue image acquisition and preprocessing

**Fig. 3:**
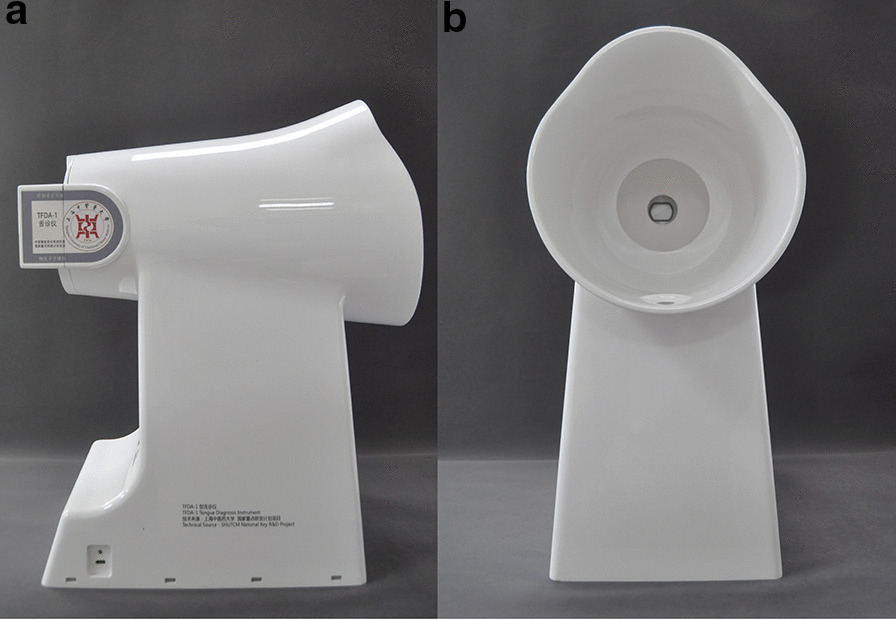
TFDA-1 tongue diagnosis instrument. **a** Side view of the instrument. **b** Front view of the instrument

To make a relatively stable tongue image dataset, tongue images were collected by uniform equipment (TFDA-1, equipment number: ER17005-201809) developed by the Shanghai University of TCM [34]. This equipment has been applied for a medical device registration certificate and mainly includes CCD equipment, standard D50 light sources, hoods, bases, and curved reflectors (see Fig. 3). The colour temperature of the LED lamp is 5003 K, and the colour rendering index is 97. The device has a high colour rendering index LED light source, and a curved reflector is set in front of it to ensure the uniformity of the illumination of each part when the tongue image is collected, which effectively ensures the stability and authenticity of the tongue image collection process.

Then, all tongue images were classified as good quality or poor quality by ten professional TCM practitioners (with over 10 years of clinical and TCM teaching experience) from the Shanghai University of TCM and its affiliated hospitals. All professionals had either corrected to normal or normal vision and reported normal colour vision. The tongue IQA was completed uniformly using an Apple Cinema HD Display (27 in., screen resolution 1920 × 1200) in the Intelligent Diagnostic Technology Laboratory of the Shanghai University of TCM.

Three steps were performed in this study to ensure the interpretation principles of tongue image quality. First, professionals unanimously agreed with the tongue image diagnosis criteria for good quality and poor quality. Second, at least 8 of 10 experts confirmed that the same label was included in the Dataset 1, and all 2531 tongue images were classified into ‘‘good” or ‘‘poor” folders by two professionals. The other eight professionals checked the labelled folders. Third, if inconsistency occurred, the corresponding tongue images were not included in this study. Only tongue images with unanimous agreement were included in the dataset for building the CNN model.

**Fig. 4:**
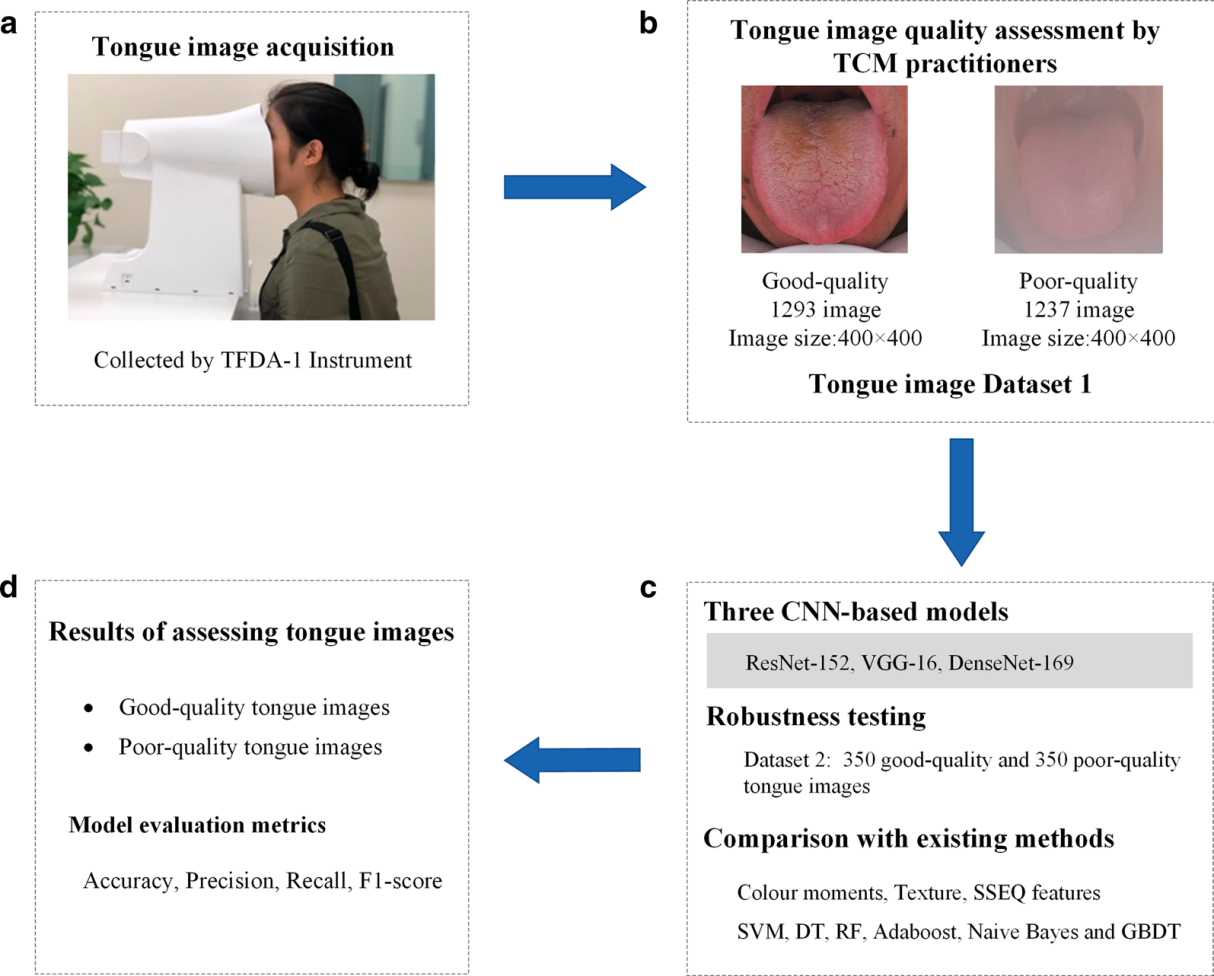
Overview of tongue IQA. **a** Example of tongue image acquisition with a uniform TFDA-1 instrument. **b** Tongue image dataset construction and examples of good-quality and poor-quality tongue images. **c** Three classical CNN models, robustness testing, and comparison with existing methods. **d** performance of assessing tongue image quality

According to the above interpretation principles, the Dataset 1, containing 1238 poor-quality tongue images and 1293 good-quality tongue images, was constructed. Among them, 1238 poor-quality images were captured in clinical research centres, including 189 cases of underexposure, 192 cases of overexposure, 168 cases of fogging, 190 cases of light leakage, 146 cases of blurred focus, 197 cases of tongue posture errors, and 156 cases of stained tongue coating. The remaining 1293 tongue images with no fogging, no underexposure, and no overexposure were selected as good-quality tongue images. The raw tongue image size was 5568 × 3711 pixels. In addition, to control the noise of the face and background areas around the tongue region, all available raw tongue images were isolated and cropped manually to the same size (400 × 400 pixels) as the tongue region before model training. Finally, we constructed the dataset, including good-quality tongue region images and poor-quality tongue region images. A schematic of the process of acquired raw tongue image Dataset 1 construction and tongue region image preprocessing is shown in Fig. 4.

### Development of CNN models

#### CNN model architecture

To prove the effectiveness of the CNN model in the present research, three classical CNN were conducted.

**Fig. 5:**
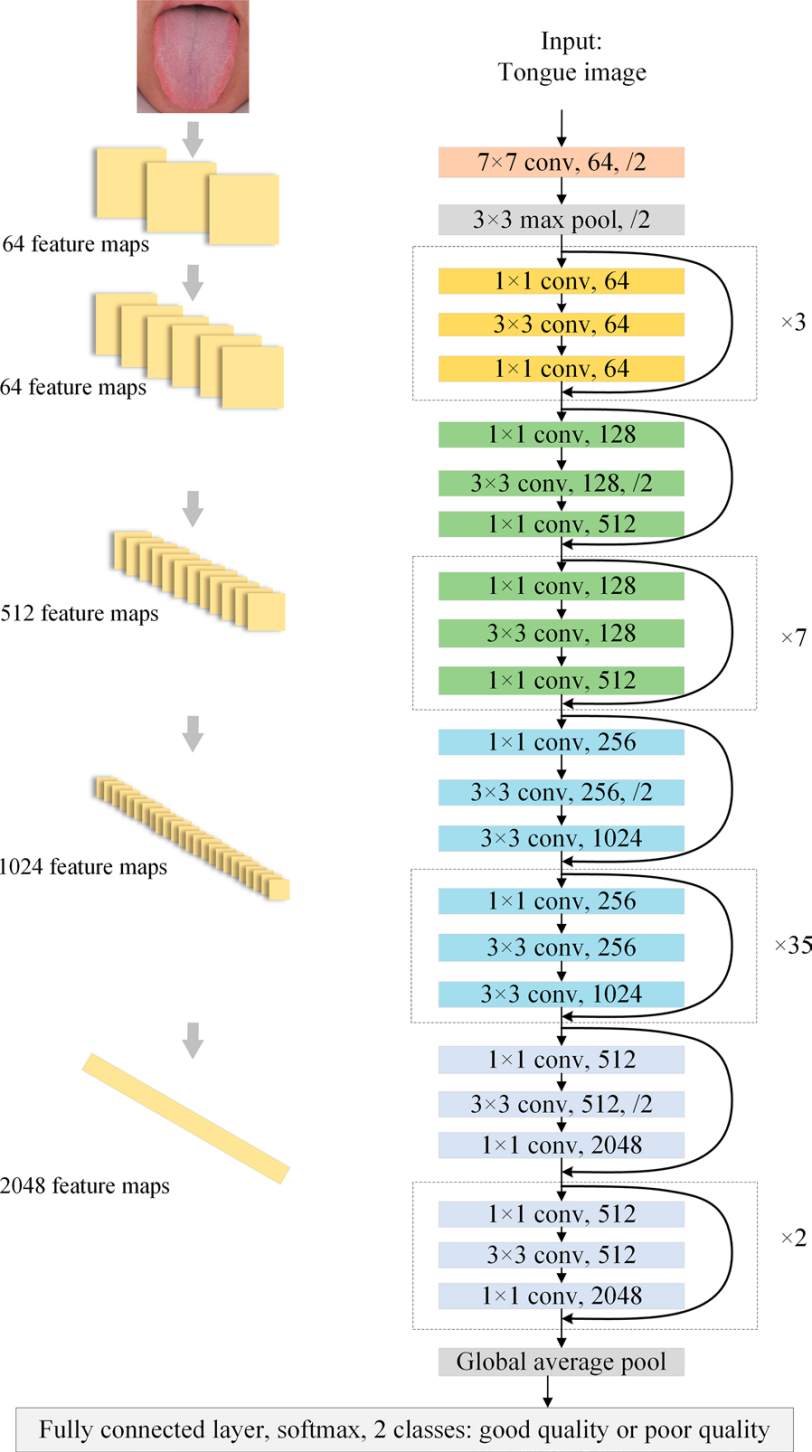
Overview of the ResNet-152 architecture for assessing tongue image quality. “7 × 7conv, 64” means that the convolutional kernel size is 7 × 7 and the filter number is 64. “/2” indicates the stride size

First, this paper uses the deep CNN model ResNet-152 based on the residual network (residual network, ResNet) [14]. ResNet-152 is a deep CNN with 152 layers, and then through 50 building blocks, each block is 3 layers, for a total of 150 layers. The last layer is an FC layer for tongue image quality classification. This layer improves the efficiency of information dissemination by adding a shortcut connection to the nonlinear convolutional layer. The residual network increases the depth of the neural network by connecting multiple residual units. This method has improved the prediction accuracy and the training speed and performs better than the traditional neural network model. A schematic diagram of the tongue IQA based on the ResNet-152 model is shown in Fig. 5, and detailed information on the ResNet-152 structure is shown in Table 1.

**Table 1:** ResNet-152 for tongue image quality control

Layers	Feature map size	Structure
Conv1	200 × 200	7 × 7 conv, 64, stride 2
Conv2_x	100 × 100	3 × 3 max pool, stride 2
		$$\left[ {\begin{array}{*{20}l} {1 \times 1} \hfill & {{\text{conv}},} \hfill & {64} \hfill \\ {3 \times 3} \hfill & {{\text{conv}},} \hfill & {64} \hfill \\ {1 \times 1~} \hfill & {{\text{conv}},} \hfill & {256} \hfill \\ \end{array} } \right] \times 3$$
Conv3_x	50 × 50	$$\left[ {\begin{array}{*{20}l} {1 \times 1} \hfill & {{\text{conv}},} \hfill & {{\text{128}}} \hfill \\ {3 \times 3} \hfill & {{\text{conv}},} \hfill & {{\text{128}}} \hfill \\ {1 \times 1~} \hfill & {{\text{conv}},} \hfill & {{\text{512}}} \hfill \\ \end{array} } \right] \times 8$$
Conv4_x	25 × 25	$$\left[ {\begin{array}{*{20}l} {1 \times 1} \hfill & {{\text{conv}},} \hfill & {{\text{256}}} \hfill \\ {3 \times 3} \hfill & {{\text{conv}},} \hfill & {{\text{256}}} \hfill \\ {1 \times 1~} \hfill & {{\text{conv}},} \hfill & {{\text{1024}}} \hfill \\ \end{array} } \right] \times 36$$
Conv5_x	13 × 13	$$\left[ {\begin{array}{*{20}l} {1 \times 1} \hfill & {{\text{conv}},} \hfill & {{\text{512}}} \hfill \\ {3 \times 3} \hfill & {{\text{conv}},} \hfill & {{\text{512}}} \hfill \\ {1 \times 1~} \hfill & {{\text{conv}},} \hfill & {{\text{2048}}} \hfill \\ \end{array} } \right] \times 3$$
Classification Layer	1 × 1	13 × 13 global average pool
		2208D fully connected layer with ReLU
		2D fully connected layer
		Softmax

Building blocks are shown in brackets, with the number of blocks stacked. Downsampling is performed by Conv3_1, Conv4_1, and Conv5_1 with a stride of 2

**Fig. 6:**
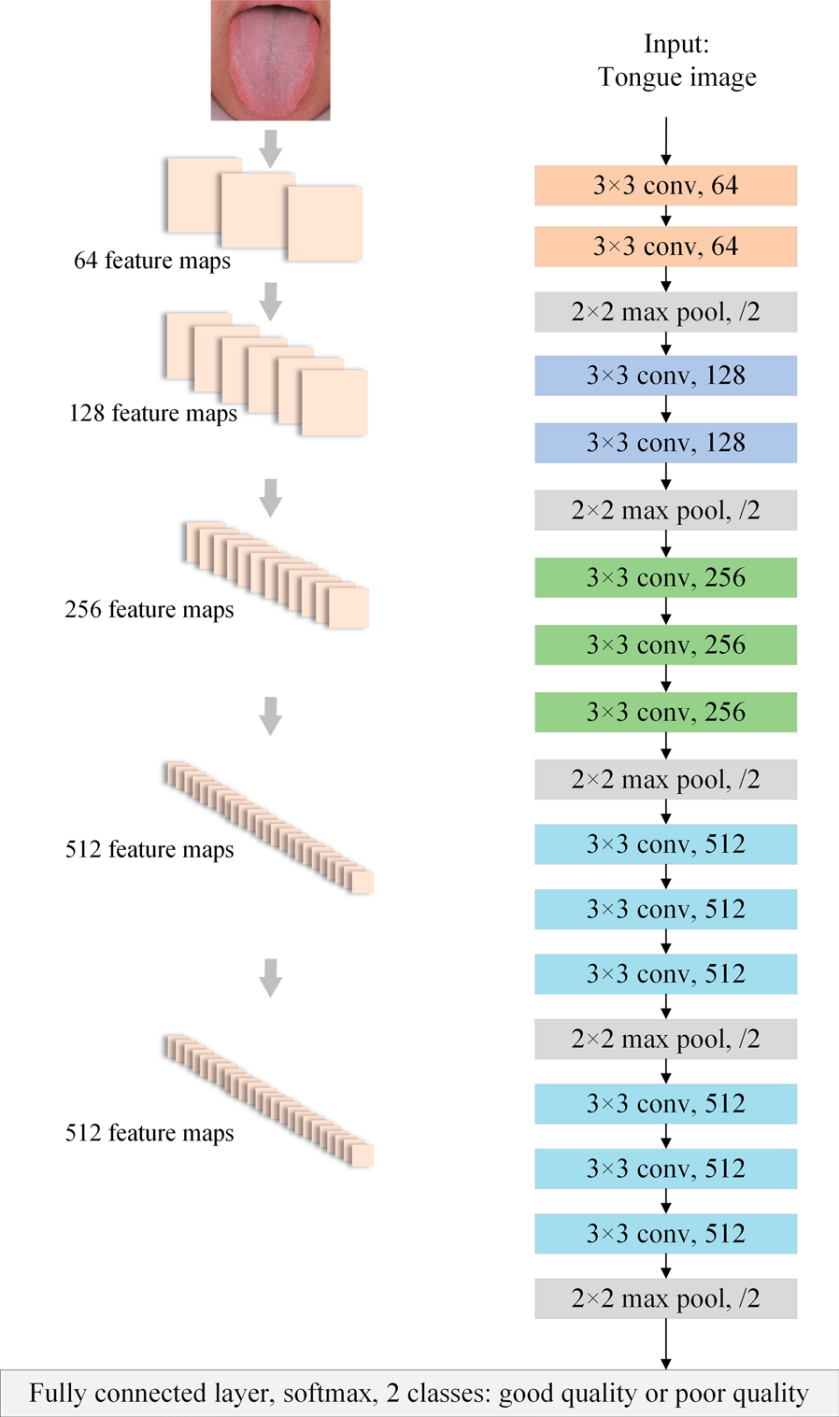
VGG-16 architecture of tongue IQA

Then, VGG-16 was used for comparative experiments. VGGNet is a deep CNN developed by researchers from the Visual Geometry Group of Oxford University and Google DeepMind [35]. VGG-16, which contains 13 convolutional layers and 3 fully connected layers, was used to improve performance by continuously deepening the network structure. For comparative analyses and reducing training time, VGG-16 was also pretrained on ImageNet datasets, and the training parameter settings were in accordance with the aforementioned ResNet-152. A schematic diagram of the tongue IQA based on VGG-16 is shown in Fig. 6, and detailed information on the VGG-16 structure is shown in Table 2.

**Table 2:** VGG-16 for tongue image quality control

Layers	Feature map size	Structure
Conv1	400 × 400	3 × 3 conv, 64
Conv2	400 × 400	3 × 3 conv, 64
Pool1	200 × 200	2 × 2 max pool, stride 2
Conv3	200 × 200	3 × 3 conv, 128
Conv4	200 × 200	3 × 3 conv, 128
Pool2	100 × 100	2 × 2 max pool, stride 2
Conv5	100 × 100	3 × 3 conv, 256
Conv6	100 × 100	3 × 3 conv, 256
Conv7	100 × 100	3 × 3 conv, 256
Pool3	50 × 50	2 × 2 max pool, stride 2
Conv8	50 × 50	3 × 3 conv, 512
Conv9	50 × 50	3 × 3 conv, 512
Conv10	50 × 50	3 × 3 conv, 512
Pool4	25 × 25	2 × 2 max pool, stride 2
Conv11	25 × 25	3 × 3 conv, 512
Conv12	25 × 25	3 × 3 conv, 512
Conv13	25 × 25	3 × 3 conv, 512
Pool5	12 × 12	2 × 2 max pool, stride 2
Classification Layer	1 × 1	2208D fully connected layer with ReLU
		2D fully connected layer
		Softmax

**Fig. 7:**
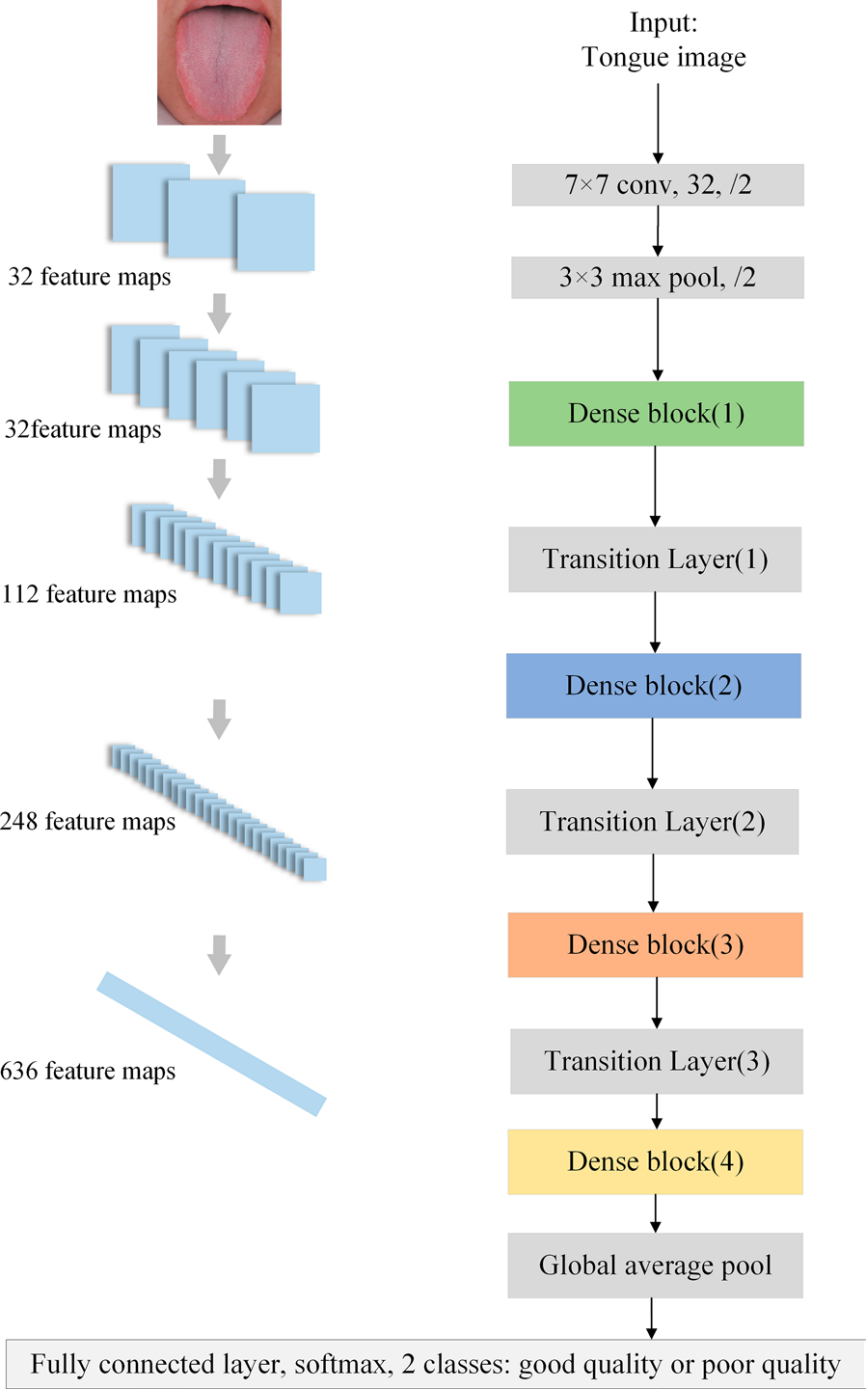
DenseNet-169 architecture of tongue IQA

**Fig. 8:**
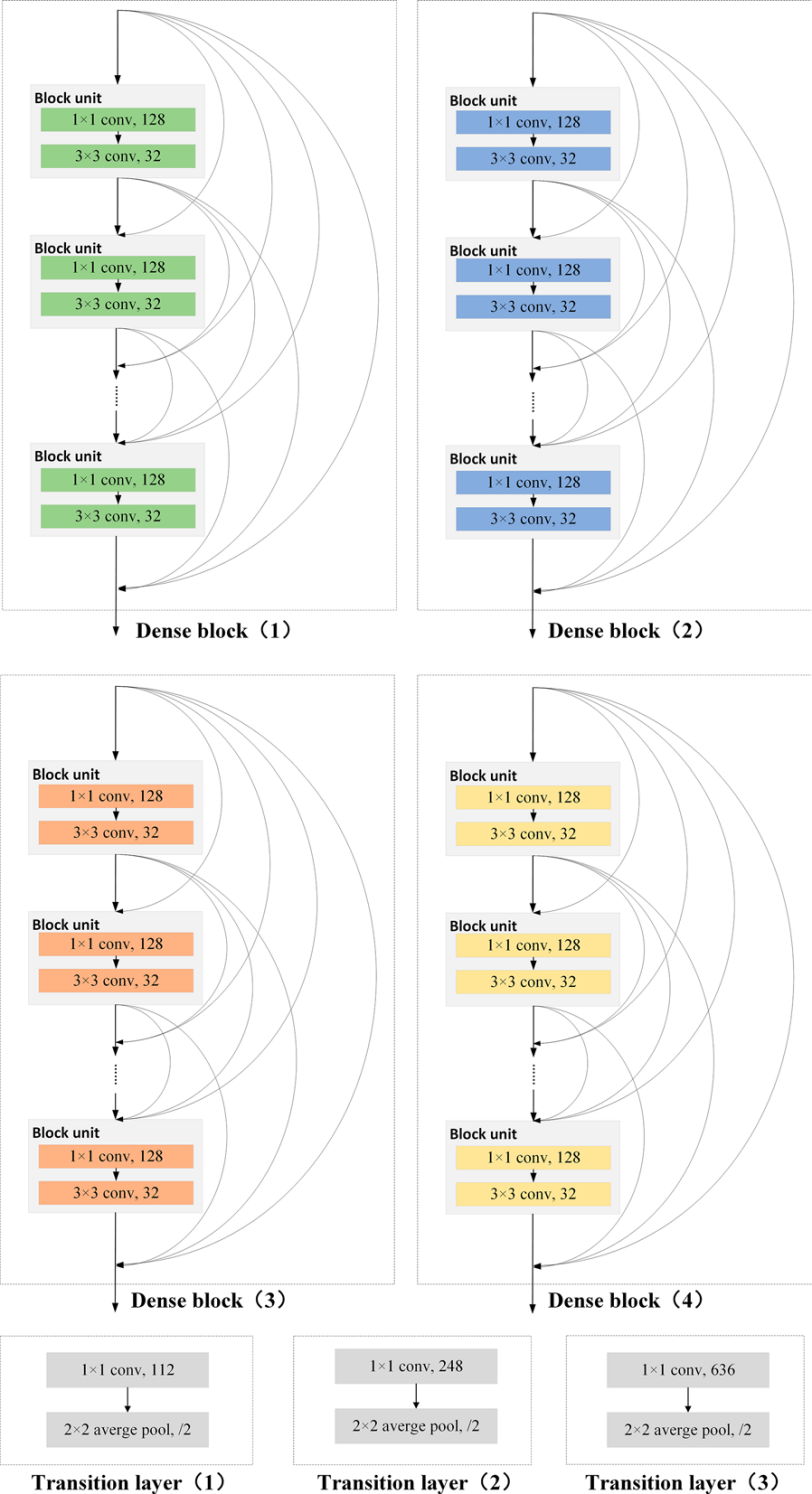
Details of the dense block and transition layer in DenseNet-169. Dense block (1), dense block (2), dense block (3), and dense block (4) contain 6, 12, 32, and 32 block units, respectively

Finally, DenseNet-169 was also used for comparative experiments. Crucially, in contrast to ResNets, DenseNets bypass signals from one layer to the next via identity connections that combine features by concatenating them. Due to the design of the dense connectivity pattern with dense blocks and transition layers, DenseNets also alleviate the vanishing gradient problem and achieve high performance in competitive object recognition benchmark tasks [36]. DenseNet-169 was also pretrained on ImageNet datasets. A schematic diagram of the tongue IQA based on DenseNet-169 is shown in Figs. 7 and 8, and detailed information on the DenseNet-169 structure is shown in Table 3.

**Table 3:** DenseNet-169 for tongue image quality control

Layers	Feature map size	Structure
Convolution	200 × 200	7 × 7 conv, 32, stride 2
Pooling	100 × 100	3 × 3 max pool, stride 2
Dense Block (1)	100 × 100	$$\left[ {\begin{array}{*{20}l} {1 \times 1} \hfill & {{\text{conv}},} \hfill & {128} \hfill \\ {3 \times 3} \hfill & {{\text{conv}},} \hfill & {32} \hfill \\ \end{array} } \right] \times 6$$
Transition Layer (1)	100 × 100	1 × 1 conv, 112
	50 × 50	2 × 2 average pool, stride 2
Dense Block (2)	50 × 50	$$\left[ {\begin{array}{*{20}l} {1 \times 1} \hfill & {{\text{conv}},} \hfill & {128} \hfill \\ {3 \times 3} \hfill & {{\text{conv}},} \hfill & {32} \hfill \\ \end{array} } \right] \times 12$$
Transition Layer (2)	50 × 50	1 × 1 conv, 248
	25 × 25	2 × 2 average pool, stride 2
Dense Block (3)	25 × 25	$$\left[ {\begin{array}{*{20}l} {1 \times 1} \hfill & {{\text{conv}},} \hfill & {128} \hfill \\ {3 \times 3} \hfill & {{\text{conv}},} \hfill & {32} \hfill \\ \end{array} } \right] \times {\text{32}}$$
Transition Layer (3)	25 × 25	1 × 1 conv, 636
	12 × 12	2 × 2 average pool, stride 2
Dense Block (4)	12 × 12	$$\left[ {\begin{array}{*{20}l} {1 \times 1} \hfill & {{\text{conv}},} \hfill & {128} \hfill \\ {3 \times 3} \hfill & {{\text{conv}},} \hfill & {32} \hfill \\ \end{array} } \right] \times {\text{32}}$$
Classification Layer	1 × 1	12 × 12 global average pool
		2208D fully connected layer with ReLU
		2D fully connected layer
		Softmax

In DenseNet-169, the growth rate is set to 32, each bottleneck layer produces 128 feature maps, and the reduction is 0.5

#### CNN model training, validation and testing

Three convolution neural networks, including VGG-16, DenseNet-169 and ResNet-152, were separately deployed to conduct experiments of classifying poor-quality tongue images and good-quality tongue images. Each model was pretrained over the dataset ImageNet to obtain initialized weights. Each well-pretrained model was employed to perform training-validation-testing experiments over Dataset 1. In each experiment, Dataset 1 was randomly divided into training set, validation set and test set according to a ratio of 8:1:1. When training model, parameters were adjusted to obtain a trained model with best performances. The adjusted parameters included epochs, batch size, optimizer, learning rate, momentum, and loss function. Also, the learning rate was set to be decreasing along with training steps. A group of model parameters with the best validation accuracy was selected, and it was set on the corresponding model to conduct 10 experiments. Performance results in the 10 experiments were averaged as the model performance. For models of VGG, ResNet and DenseNet, their parameters settings were the same. And the parameters settings were epochs of 30, optimizer of stochastic gradient descent optimization (SGD), learning rate of 0.01, momentum of 0.9, batch size of 32, and Cross Entropy loss. CNN models were trained using PyTorch (version 1.3.1) and Python (version 3.6) frameworks on the Ubuntu system (version 14.04). CNN models were developed on the hardware DEVTOP AIX4750 produced by OMNISKY with 4 NVIDIA GTX 1080Ti GPU, i7-6850 K CPU, 64 GB DDR4 RAM, and 512 GB SDD.

#### CNN model testing on new dataset

Testing was performed in new dataset (Dataset 2) by other clinical research centres with different kinds of tongue diagnosis instruments. A new testing dataset including 700 tongue images acquired by the TFDA-1 and TDA-1 instruments was constructed. (These instruments use different CCDs and illumination.) In addition, we cropped each raw tongue image into a tongue region image of the same size. The tongue image dataset was also classified into 350 poor-quality tongue images and 350 good-quality tongue images by the same professionals as mentioned above in “Tongue image acquisition and preprocessing” section. The 350 poor-quality tongue images included 7 categories as mentioned in “Background” section. Then, all tongue images Dataset 2 here were classified using the aforementioned 3 CNN models trained by dataset1.

### Comparison with existing methods

According to Zhang’s method [12, 13], 350 good-quality tongue images and 350 poor-quality tongue images were randomly selected to form Dataset 3 from Dataset 1. In each experiment, Dataset 1 was randomly splitted into training set, validation set and test set according to a ratio of 7:1.5:1.5. First, colour features, texture features and SSEQ features were extracted to evaluate the quality of tongue image. Colour moments are computed as features in tongue IQA, including 3 dimensional features: the first colour moment “mean”, the second colour moment “standard deviation”, and the third colour moment “skewness”. The coarseness and contrast of the tongue image were computed as 2 dimensional texture features. All 700 tongue images were decomposed into low, middle and high scales and then 6 dimensional spatial entropy features and 6 dimensional spectral entropy features were extracted as SSEQ features [37–39]. Then, all 17 dimensional extracted features were normalized to [0,1] and fed into 6 machine learning classifier SVM, Decision Tree, Random Forest, Naïve Bayes, Adaboost and GBDT for the binary classification of tongue IQA, respectively.

For comparison, we used ResNet-152, VGG-16, and DenseNet-169 to construct model on Dataset 3. The parameters settings were the same aforementioned in “CNN model training, validation and testing” section. It was set on the corresponding model to conduct 10 experiments and performance results in the 10 experiments were averaged as the model performance. The main procedures are shown in Fig. 4.

### Model evaluation metrics

Accuracy is one of the most commonly used model evaluation metrics in machine learning. It indicates the average classification effect describing the overall performance of all categories. In addition, this study also uses three metrics, namely, precision, recall, and F1-score, to evaluate and analyse the performance of the model. The accuracy (Eq. (1)), precision (Eq. (2)), recall (Eq. (3)) and F1-score (Eq. (4)) were used to evaluate the performance of the CNN model [40–43]. True positive (TP) represents the number of images correctly classified as poor-quality tongue images, true negative (TN) represents the number of images correctly classified as good-quality tongue images, false positive (FP) represents the number of images incorrectly classified as poor-quality tongue images, and false negative (FN) represents the number of images incorrectly classified as good-quality tongue images. These parameters compose, therefore, a complementary metric to the overall accuracy. Macro-averaging is used for models with more than 2 target classes. Macro-averaging is performed by first computing the precision, recall, and F1-score of each class and then taking the average of all precision and recall values and F1-scores. For each of the three CNN models, ten experiments were performed in a fixed parameter setting. The average and the standard deviation were calculated over the results in the ten experiments for each kind of metric.

1 $$Accuracy = \frac{TP + TN}{{TP + FP + TN + FN}}$$

2 $$Precision = \frac{TP}{{TP + FP}}$$

3 $$Recall = \frac{TP}{{TP + FN}}$$

4 $$F1 - score = \frac{2 \times Precision \times Recall}{{Precision + Recall}}.$$

## Results

### Testing results on the tongue image dataset with ResNet-152

The accuracy of the model in the training set and the validation set is close to 100%, and the training loss gradually decreases as the epoch increases. The tongue image quality classification results by the ResNet-152 architecture on 2531 raw tongue images are shown in Table 4. As expected, the classification performance of the ResNet-152 model remains stable and satisfactory. The macro-averaged accuracy is 98.82%, which proves the effectiveness of the CNN method. The macro precision is 98.83%, and the macro recall is 98.81%, revealing that the ResNet-152 models have a relatively high level of precision and recall.

**Table 4:** Results of the ResNet-152 architecture

Folds	Precision	Recall	F1-score	Accuracy
Fold 1	99.21%	99.21%	99.21%	99.21%
Fold 2	98.04%	98.02%	98.03%	98.03%
Fold 3	98.45%	98.41%	98.43%	98.43%
Fold 4	98.83%	98.81%	98.82%	98.82%
Fold 5	98.42%	98.42%	98.42%	98.43%
Fold 6	98.81%	98.83%	98.82%	98.82%
Fold 7	99.21%	99.21%	99.21%	99.21%
Fold 8	98.83%	98.81%	98.82%	98.82%
Fold 9	99.24%	99.19%	99.22%	99.21%
Fold 10	99.24%	99.19%	99.22%	99.21%
Average (SD)	98.83% (0.42%)	98.81% (0.42%)	98.82% (0.42%)	**98.82%** (0.41%)

The bold value mean average accuray results of ten folds

### Comparison with VGG-16 and DenseNet-169

To further study whether the CNN architecture may influence the experimental results, VGG-16 and DenseNet-169 were used for comparison analysis. The results are shown in Tables 5 and 6. The macro-averaged accuracy is 96.89% by VGG-16 and 98.82% DenseNet-169 on the same testing tongue image subset. However, the ResNet-152 and DenseNet-169 architectures can increase the accuracy of poor-quality tongue image classification by nearly 2% on the same testing tongue image subset. As we expected, the macro-averaged precision is 96.91% and recall is 96.88% by VGG-16, and the macro-averaged precision is 98.83% and recall is 98.82% by DenseNet, indicating that the models have impressively high precision and recall.

**Table 5:** Results of the VGG-16 architecture

	Precision	Recall	F1-score	Accuracy
Fold 1	95.27%	95.27%	95.27%	95.28%
Fold 2	96.50%	96.43%	96.47%	96.46%
Fold 3	95.35%	95.24%	95.29%	95.28%
Fold 4	96.84%	96.87%	96.86%	96.85%
Fold 5	96.85%	96.85%	96.85%	96.85%
Fold 6	98.04%	98.02%	98.03%	98.03%
Fold 7	98.04%	98.02%	98.03%	98.03%
Fold 8	96.09%	96.04%	96.07%	96.06%
Fold 9	98.45%	98.41%	98.43%	98.43%
Fold 10	97.63%	97.66%	97.64%	97.64%
Average (SD)	96.91% (1.13%)	96.88% (1.14%)	96.89% (1.13%)	**96.89%** (1.14%)

The bold value mean average accuray results of ten folds

**Table 6:** Results of the DenseNet-169 architecture

	Precision	Recall	F1-score	Accuracy
Fold 1	99.21%	99.23%	99.22%	99.21%
Fold 2	98.83%	98.81%	98.82%	98.82%
Fold 3	98.08%	98.00%	98.04%	98.03%
Fold 4	97.64%	97.64%	97.64%	97.64%
Fold 5	98.81%	98.83%	98.82%	98.82%
Fold 6	98.87%	98.79%	98.83%	98.82%
Fold 7	99.21%	99.23%	99.22%	99.21%
Fold 8	99.21%	99.21%	99.21%	99.21%
Fold 9	98.81%	98.83%	98.82%	98.82%
Fold 10	99.60%	99.62%	99.61%	99.61%
Average (SD)	98.83% (0.58%)	98.82% (0.60%)	98.82% (0.59%)	**98.82%** (0.59%)

The bold value mean average accuray results of ten folds

### Robustness testing

To further evaluate the robustness of our CNN models, we also conducted comparative experiments on the new testing dataset. The new tongue image dataset consisted of 350 poor tongue images from different tongue diagnosis instruments. The overall accuracy of the trained CNN models is 97.71% with VGG-16, 99.04% with ResNet-152, and 98.89% with DenseNet-169 for the same testing dataset (Table 7). In addition, since the tongue images from this testing dataset were acquired under different light conditions, the macro accuracy of the testing new dataset is also higher than 97%, revealing that the CNN models have good robustness and can be generalized to images from different instruments with various CCDs and illuminations.

**Table 7:** Testing results on Dataset 2

Model	Precision	Recall	F1-score	Accuracy
VGG-16	97.77% (0.47%)	97.71% (0.51%)	97.74% (0.49%)	**97.71%** (0.51%)
ResNet-152	99.05% (0.20%)	99.04% (0.20%)	99.05% (0.20%)	**99.04%** (0.20%)
DenseNet-169	98.89% (0.17%)	98.89% (0.18%)	98.89% (0.17%)	**98.89%** (0.18%)

The bold values mean average accuray results of ten folds

**Fig. 9:**
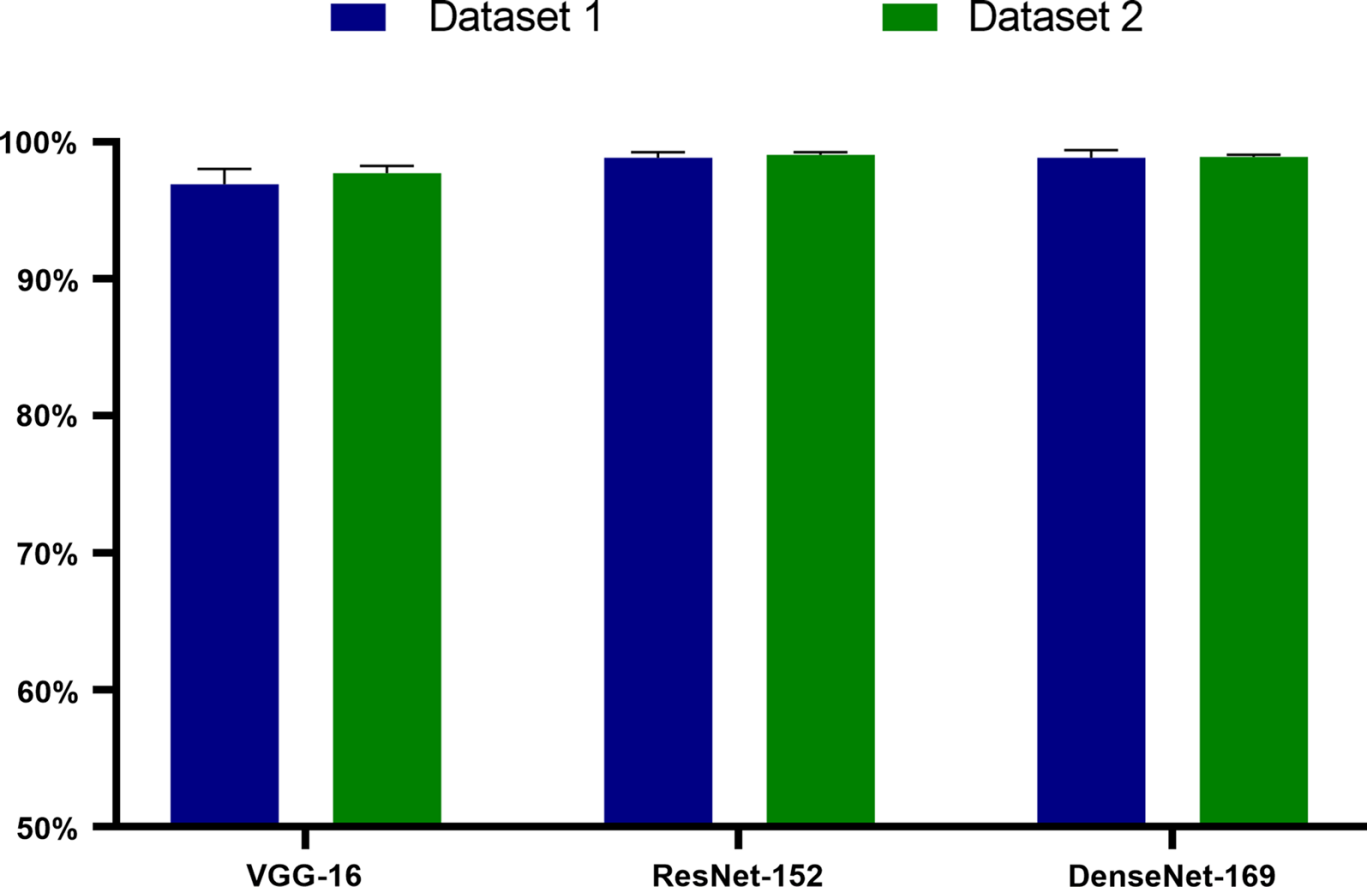
Macro-averaged accuracy of the tongue image quality classification model. Our CNN models with ResNet-152 and DenseNet-169 can increase the accuracy of poor-quality tongue image classification by nearly 2%

The average accuracy results over different datasets are shown in Fig. 9 for ResNet-152, VGG-16, and DenseNet-169 with different CNN models. The tongue IQA model based on ResNet-152 obtained the best testing results, with an average accuracy of 99.04%, precision of 99.05%, recall of 99.04%, and F1-score of 99.05%, as shown in Fig. 9.

### Results of comparison with existing methods

**Fig. 10:**
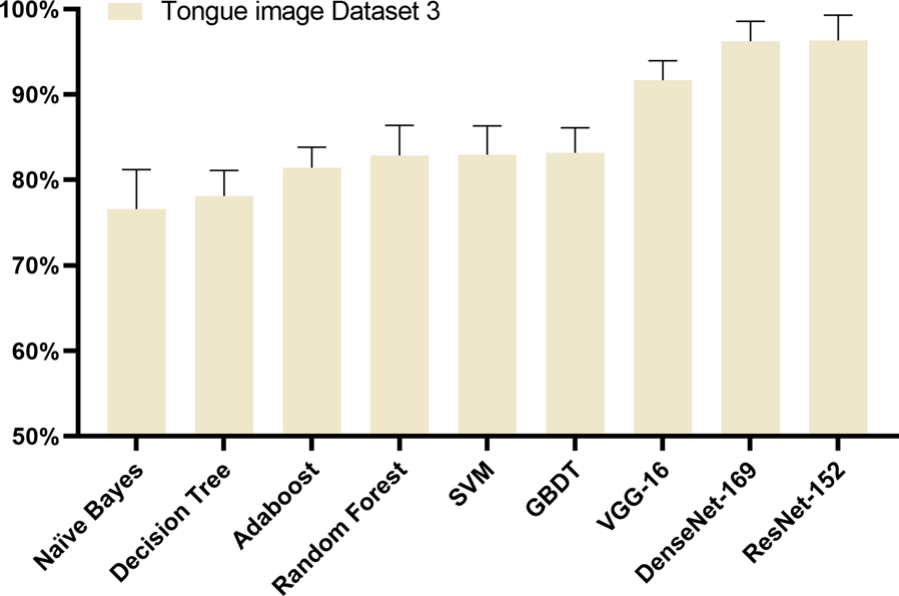
Classification accuracy results of the tongue IQA model over Dataset 3. Comparison with Zhang’s methods, the model ResNet-152 improve the accuracy of classifying poor-quality versus good-quality tongue images by nearly 13%

The results show that the GBDT model achieved the best accuracy of 83.15%, followed by SVM with accuracy of 82.95%, Random Forest with accuracy of 82.84%, Adaboost with accuracy of 81.42%, and Decision Tree with accuracy of 78.09%. The performance of Naïve Bayes classification was the worst, with an accuracy of 76.57%. The classification performance of the three CNN models is significantly better than Zhang’s methods. The overall classification accuracy over Dataset3 by using VGG-16 was 91.68%, the accuracy of 96.22% for DenseNet-169, and the accuracy of 96.32% for ResNet-152, as shown in Table 8. The experimental results in Table 8 and Fig. 10 indicated that ResNet-152 outperformed the models in classifying good-quality versus poor-quality tongue images, including VGG-16, SVM, RF, and GBDT.

**Table 8:** Comparison with existing methods

Model	Precision	Recall	F1-score	Accuracy
SVM	83.39% (7.00%)	83.25% (4.32%)	83.06% (3.23%)	82.95% (3.20%)
Decision Tree	79.52% (4.41%)	76.39% (6.74%)	77.66% (3.86%)	78.09% (2.86%)
Random Forest	84.10% (6.76%)	81.67% (4.51%)	82.64% (3.70%)	82.84% (3.35%)
Naïve Bayes	88.86% (6.15%)	61.24% (7.15%)	72.26% (5.88%)	76.57% (4.39%)
Adaboost	83.50% (4.60%)	79.23% (6.70%)	81.00% (2.74%)	81.42% (2.26%)
GBDT	84.46% (4.82%)	81.72% (5.61%)	82.86% (3.36%)	83.15% (2.79%)
VGG-16	91.87% (2.14%)	91.68% (2.16%)	91.77% (2.15%)	91.68% (2.16%)
ResNet-152	96.36% (2.81%)	96.32% (2.81%)	96.34% (2.81%)	96.32% (2.81%)
DenseNet-169	96.30% (2.19%)	96.22% (2.23%)	96.26% (2.21%)	96.22% (2.23%)

Based on the experimental results of the three CNN models and the above comparisons, it is concluded that the three CNN models can be used for screening image quality over massive tongue images.

## Discussion

Tongue inspection is an important objective diagnostic method in the process of TCM clinical diagnosis and treatment. The characteristics of tongue signs are important information sources for TCM clinical pattern identification and treatment, which is of great significance for the discrimination of cold, heat, deficient and excessive patterns and the treatment of medication. It is a common consensus that standardized tongue image acquisition criteria are important for objective tongue diagnosis in TCM clinical research. The quality of tongue images is a crucial indicator in artificial intelligent tongue diagnosis systems. Limited by the individual differences of the operators of the tongue diagnosis instruments, in the process of advancing the "Research and Development of the Intelligent Tongue Diagnosis System" project, we found that even though we have already conducted multiple standardized tongue image collection trainings, the obtained tongue image quality is still unqualified, and yet there are many poor-quality tongue images mentioned in “Background” section.

The quality control of tongue images is the preliminary work of constructing the standard dataset of tongue images of TCM, especially during the remote diagnosis and treatment of the Chinese medicine Internet. If poor-quality images in Fig. 2 are mixed in, these may lead to the wrong diagnosis, so an efficient and intelligent tongue image quality screening model is urgently needed. It is difficult for traditional pattern recognition methods to quickly identify a variety of poor-quality tongue images.

The purpose of this study was to solve the problem of automatically controlling the quality of a large-sample tongue image database. The advantage of deep learning algorithms, especially CNNs, lies in their powerful feature extraction capabilities. It is possible to discover important hierarchical relationships in the data through algorithms without laboriously crafting features.

To our knowledge, this is the first study using deep CNNs for assessing tongue image quality. This is also the first study to put forward and systematically summarize the quality control of tongue images, and the performances of three classical deep learning models, which were used to automatically identify tongue images with good quality and poor quality, were compared.

First, we collected 2531 raw tongue images by uniform instruments and categorized these tongue images into 1238 poor-quality tongue images and 1293 good-quality tongue images by 10 TCM professionals. We also preprocessed the tongue images to the same size and accumulated a Dataset 1. We used different CNN models, namely, ResNet-152, VGG-16, and DenseNet-169, to extract features and perform binary classifications.

Then, we collected 700 tongue images (Dataset 2) from other clinical research centres by different instruments to verify our CNN models. Interestingly, the macro-averaged accuracy of the CNN models was impressively over 96% both on Dataset 1 and Dataset 2. Moreover, ResNet-152 and DenseNet-169 achieved a better classification accuracy than VGG-16, mainly due to the greater depths and powerful feature extraction capabilities of the networks. Even in the new dataset, our models with ResNet-152, VGG-16, and DenseNet-169 can achieve macro-averaged accuracy, precision, and recall values and F1-scores exceeding 98%. This indicates that the CNN models can be effective and adaptable to tongue images acquired by instruments with different illuminations and CCDs.

Finally, colour moments, texture features, SSEQ features were extracted from Dataset3 and were fed into SVM, Decision Tree, Random Forest, Naïve Bayes, Adaboost for tongue IQA [12, 13]. The results showed that the GBDT model was with the best accuracy of 83.15%, followed by SVM with accuracy of 82.95%, Random Forest with accuracy of 82.84%, which was a little less than the reported accuracies in the literatures of [12, 13]. One possible reason is that Zhang's method was proposed for recognizing two types of poor-quality tongue images, i.e., unsuitable posture and blurry. Foggy, underexposure, overexposure, moss staining and other poor-quality types were not considered. Therefore, the three extracted features cannot fully cover the types of poor-quality tongue images. Comparison with Zhang’s methods, ResNet-152 can improve the accuracy of classifying poor-quality versus good-quality tongue images by nearly 13%. The results showed that ResNet-152 can well capture features of poor-quality images. However, the overall classification accuracy over Dataset 3 is smaller than that on Dataset 1. The sample size of Dataset 3 is smaller, being one fourth of the sample size of Dataset 1. The small sample size possibly restricts the generalizing capability of the trained CNN models.

In summary, these testing and comparison results demonstrate that the CNN models in the present study performed impressively in the classification of poor-quality tongue images. The experimental results showed that the accuracy of these three deep learning models was over 96%, and the accuracy of ResNet and DenseNet was over 98%. The results showed that it is feasible to apply the depth CNN model to the quality control of tongue images and that the practicability of this research work provides a preliminary research basis for establishing the standard dataset of tongue images in the future.

With the assistance of deep learning methods, the proposed CNN method on our tongue image dataset for binary classification exhibits especially high accuracy, so the tongue IQA can be easily achieved. This provides reliable premises, guarantees the stability of later data analysis, and meets the clinical research needs of tongue diagnosis.

At this stage, this study did not establish a new model for tongue image quality control and did not use a recent state-of-the-art CNN model. According to the literature we consulted, there was no open-source dataset for testing in the field of tongue diagnosis of TCM. Thus, we focused on the quality control of tongue images to build an open-source tongue image database, ensure the quality of pictures in the tongue image database, and provide reliable data support for intelligent technology research on tongue diagnosis in TCM. Moreover, there are still several shortcomings in this study. The quality assessment of tongue images can be further improved in the future.

First, with the training of standardized acquisition techniques, subjects should be given sufficient guidance before collecting tongue images. Poor-quality tongue images originating from the operators can be avoided.

Second, considering the good performance of CNN models and the problem of poor interpretability of CNNs [44], some scholars have also conducted visual analysis and research on the process of convolution, pooling, and prediction classification of CNNs [45, 46]. Our results show that the deeper architectures (ResNet-152, DenseNet-169) outperformed the shallower architecture (VGG-16) for all the evaluation metrics, including accuracy, precision, recall, and F1-score. However, the problem usually becomes more computationally intensive when the CNN layer becomes deeper. Therefore, to balance the computational cost and model performance well, it is essential to develop lighter CNN models for tongue image quality.

Third, further research is required to investigate the tongue IQA model for more diverse scenes, such as smartphones [47, 48], aiming at various kinds of poor-quality tongue image appearances. Constructing multiple classification models to distinguish poor tongue quality images into more groups may increase the clinical applicability to expand the clinical application level of tongue diagnosis. In the next step, we plan to study how to use the latest image recognition technology to improve the performance of automatic tongue image recognition, with the aim to establish tongue image quality control suitable for more scenes.

## Conclusions

Our research findings demonstrate various CNN models in the decision-making process for the selection of tongue IQA and indicate that applying deep learning methods, specifically deep CNNs, to evaluate poor-quality tongue images is feasible.

## Data Availability

The datasets used and/or analyzed during the current study are available from the corresponding author on reasonable request.

## Abbreviations

IQA: Image Quality Assessment
CNN: Convolutional Neural Network
Acc: Accuracy
Rec: Recall
Pre: Precision
SD: Standard Deviation
FC layer: Fully Connected Layer
Conv: Convolution
ReLU: Rectified Linear Unit
SVM: Support Vector Machine
RF: Random Forest
GBDT: Gradient Boosting Decision Tree
Adaboost: Adaptive Boosting Algorithm
DT: Decision Tree
